# Toxic and Nutritional Optic Neuropathies—An Updated Mini-Review

**DOI:** 10.3390/ijerph19053092

**Published:** 2022-03-06

**Authors:** Jacek Baj, Alicja Forma, Joanna Kobak, Magdalena Tyczyńska, Iga Dudek, Amr Maani, Grzegorz Teresiński, Grzegorz Buszewicz, Jacek Januszewski, Jolanta Flieger

**Affiliations:** 1 Department of Human Anatomy, Medical University of Lublin, Jaczewskiego 4, 20-090 Lublin, Poland; jacek.baj@umlub.pl (J.B.); amrmaanni@gmail.com (A.M.); 2 Department of Forensic Medicine, Medical University of Lublin, Jaczewskiego 8b, 20-090 Lublin, Poland; kobak.joannaelzbieta@gmail.com (J.K.); m.tyczynska@onet.pl (M.T.); iga.dudek6@gmail.com (I.D.); grzegorzteresinski@umlub.pl (G.T.); g.buszewicz@umlub.pl (G.B.); jacek.januszewski000@gmail.com (J.J.); 3 Department of Analytical Chemistry, Medical University of Lublin, Chodźki 4A, 20-093 Lublin, Poland; j.flieger@umlub.pl

**Keywords:** toxic optic neuropathy, alcohol, tobacco, heavy metals, drugs, nutritional deficiencies

## Abstract

Optic neuropathies constitute a group of conditions with various etiologies and might be caused by different factors; we can distinguish the genetic and acquired causes of optic neuropathies. Even though the symptoms are not highly specific, this condition is primarily characterized by unilateral or bilateral vision loss with worsening color detection. The loss may be acute or gradual depending on the causation. In this article, we included a specification of toxic optic neuropathy (TON) mainly triggered by alcohol abuse and also the usage of other substances, including drugs or methanol, as well as intoxication by metals, organic solvents, or carbon dioxide. Nutritional deficiencies, vitamin absorption disorder, and anemia, which usually appear during excessive alcohol intake, and their effect on the etiology of the optic neuropathy have been likewise discussed.

## 1. Introduction

Heavy alcohol consumption or binge drinking is known to cause severe health consequences that might even lead to death; however, light to moderate drinking might decrease the risk of cardiovascular and all-cause mortality [1,2]. Low-volume alcohol consumption reduces the risk of coronary heart disease, heart failure, type 2 diabetes mellitus, ischemic stroke, or dementia [3,4,5,6]. Paradoxically, even low to moderate alcohol ingestion is toxic to neurons, increases the risk of hemorrhagic stroke, induces liver injury, and contributes to the development of breast, oral, and gastrointestinal cancers [5,7,8,9,10,11,12,13,14]. Excessive alcohol intake is defined as the consumption of more than 20 g of pure alcohol in women or more than 40 g of pure alcohol daily in men; however, definitions often vary [15,16,17]. In addition, specific drinking patterns play a substantial role in the overall effect of alcohol intake on health [17].

It is now well established that excessive alcohol consumption leads to serious illnesses, primarily affecting the liver (especially cirrhosis), the digestive tract, and the heart and cardiovascular system (especially arrhythmias, cardiomyopathy, heart failure, hypertension, and atherosclerosis), and causes diseases of the peripheral and central nervous system [16,18,19]. Further, neoplasms such as oral cavity, pharyngeal, laryngeal, esophageal, liver, colorectal, and female breast cancers are also reported to be related to excessive alcohol intake [20]. According to a recent report by the World Health Organization (WHO), in 2016, harmful alcohol usage contributed to approximately 3 million deaths, which accounted for 5.3% of all deaths worldwide. Although globally the percentage of drinkers decreased by almost 5% between 2000 and 2016, the total per capita alcohol consumption increased among drinkers over the same time in most parts of the world [21].

## 2. The Effects of Alcohol on the Central and Peripheral Nervous Systems

Alcohol affects the central and peripheral nervous systems in both direct and indirect ways. Ethanol appears to be a direct neurotoxin, whereas indirect effects are associated with liver injury, malnutrition, vitamin deficiencies, traumas, infections, or hypoglycemia [16,22,23,24,25]. Neurological complications secondary to excessive alcohol drinking include, inter alia, Wernicke’s encephalopathy, Korsakoff syndrome, dementia, cerebellar degeneration, alcohol-related peripheral neuropathy, or alcohol-related nerve compression leading to nerve palsy [16,23,26,27,28,29,30,31].

The prevalence of alcoholic neuropathy is estimated to be 25–66% among chronic alcoholics in the United States. Most common abnormalities primarily include the sensory, motor, and autonomic nerves that are mainly associated with a weakening of axons and the thinning of the myelin sheaths [28,32]. Alcohol-related peripheral neuropathy (ALN) primarily appears as a sensorimotor, axonal, length-dependent neuropathy with dominant sensory derangements. The disturbances within the functions of the motor nerves predominantly appear at a later stage of disease [33]. Regarding length-dependent axonal degeneration, the fiber loss is more severe distally and progresses proximally, which might be associated with the impairment of the axonal transport and cytoskeletal properties by ethanol exposure [28,34]. Interestingly, ALN is associated not only with distal, but also proximal small-fiber degeneration [35]. Alcohol-induced autonomic dysfunction seems to more frequently affect the parasympathetic rather than the sympathetic nervous system and often does not present with symptoms [36]. Contrarily, patients with typical somatic ALN initially report paresthesia and pain with further loss of proprioception, gait disturbances, and loss of reflexes [37,38]. The clinical importance of alcohol-induced autonomic neuropathy arises from the relationship with an increased mortality rate, mainly associated with cardiovascular and respiratory events [38,39]. There are many studies suggesting that alcoholic neuropathy is the result of a multifactorial process, primarily involving the direct neurotoxic effect of alcohol or its metabolites, and is continually modulated by other factors, including thiamine deficiency, altered thiamine metabolism, malnutrition, or impurities of alcohol (primarily including lead, which is often found in alcohol beverages); however, the appropriate pathogenesis is still a subject of debate [22,28,35]. Additionally, genetic factors, such as mutation of the aldehyde dehydrogenase-2 (ALDH2) gene, cause the accumulation of acetaldehyde, an ethanol metabolite, whose toxic effect may contribute to the development of alcoholic polyneuropathy [33,40].

Peripheral neuropathy may be caused by numerous factors; hence, the differential diagnosis of alcoholic neuropathy should include, inter alia, endocrine diseases (diabetes mellitus, hypothyroidism), infectious diseases (HIV infection, HCV infection, syphilis, and Lyme borreliosis), paraneoplastic neuropathies, chronic liver disease, amyloidosis, poisonings (arsenic, lead, mercury, and organophosphates), the use of medications (metronidazole and isoniazid), autoimmune Guillain–Barré syndrome, or genetic diseases (e.g., Charcot–Marie–Tooth neuropathy) [37,41,42,43,44,45,46,47,48,49,50,51,52,53,54].

**Figure 1:**
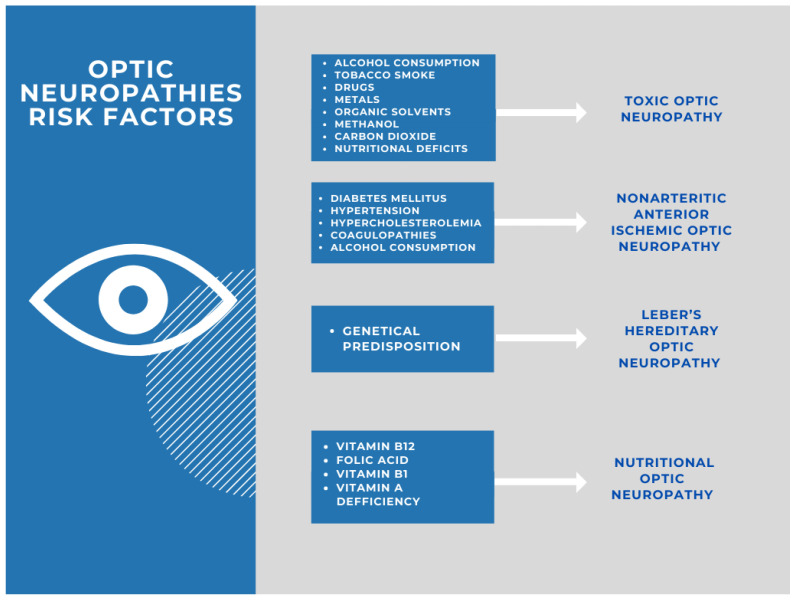
Risk factors for optic neuropathies.

Among the alcohol-related neuropathies, optic neuropathy, also known as tobacco–alcohol optic neuropathy, remains one of the most often diagnosed; however, the exact interaction or synergism of alcohol and tobacco as triggering factors has never been conclusively proven [55]. Patients may present with distinct bilateral visual disturbance, severely affected visual acuity, symmetric central or cecocentral scotomas, and acquired derangements of color vision [56]. Substantially, alcohol has no longer been recognized as a toxin that, itself, contributes to toxic optic neuropathy [57,58,59]. Alcoholism primarily leads to undernutrition through the impairments of gastrointestinal absorption and the susceptibility to consumption of smaller amount of essential nutrients and vitamins by alcohol misusers, and it appears as a risk factor of developing nutritional optic neuropathy (Figure 1) [28,58,59].

The precise pathological mechanism responsible for tobacco–alcohol optic neuropathy remains unclear; nevertheless, vitamin B12 and folate deficiencies and the cyanide in tobacco may be associated with the demyelination of the optic nerves. The cyanide and free radicals may impair the mitochondrial respiratory cycle and damage mitochondrial DNA [60]. It was shown that some patients initially diagnosed with tobacco optic neuropathy had a genetic mutation distinctive for Leber hereditary optic neuropathy (LHON)—congenital mitochondrial disease. Therefore, tobacco-induced optic neuropathy could have been misdiagnosed [55]. Nevertheless, smoking might significantly increase disease penetrance among the carrier’s mutation characteristic for LHON; similarly, heavy alcohol drinking may be a risk factor for developing LHON [61]. Due to the above-mentioned facts, presently, it is claimed that the term ‘alcohol–tobacco optic neuropathy’ is misleading and should not be used [59].

## 3. Pathophysiology of Alcohol-Induced Neuropathies

There is a great deal of controversy surrounding the pathogenesis of alcoholic neuropathy. Some researchers have considered it to be associated with nutritional deficiencies, mostly thiamine deficiency [62,63]. There is a close association between thiamine deficiency and chronic alcoholism [62]. It is worth mentioning that thiamine deficiency might induce neuropathy in people who chronically consume excessive amounts of alcohol [64]. The intestinal absorption of thiamine is diminished by ethanol [64]. The same also reduces the amount of thiamine stored in the liver while also affecting thiamine phosphorylation [65]. Additionally, chronic alcoholics tend to consume very small amounts of vitamins as well as essential nutrients [63,64]. Nutritional absorption by their gastrointestinal tract is also impaired. It is worth mentioning that these relationships are the reason why chronic alcoholism is considered to be a major risk factor for the deficiency of thiamine.

Additionally, studies have shown that ethanol and its metabolites have a direct neurotoxic effect on the body system. Bosch et al. [66] found that there was an intense axonal degeneration in rats receiving ethanol. Studies involving humans have also shown that ethanol has a direct toxic effect on numerous systems, primarily including the central and peripheral nervous systems [67,68]. This is important because a dose-dependent relationship has been established between the severity of neuropathy and a lifetime intake of alcohol [67,68]. The mechanism behind alcoholic neuropathy is yet not fully understood. However, a couple of explanations have been presented, including the activation of microglia of the spinal cord after the chronic consumption of alcohol [69], oxidative stress that results in nerve damage by free radicals, pro-inflammatory cytokine release coupled with protein kinase C activation [70], the actions of classical mitogen-activated protein kinases or extracellular signal-regulated kinases [71], and the involvement of the hypothalamo–pituitary–adrenal system [72] and the opioidergic system [69].

According to several studies, the chronic intake of alcohol can trigger a decrease in the nociceptive threshold and increase the release of the pro-inflammatory cytokines and oxidative–nitrosative stress alongside protein kinase C activation [70,71]. As such, alcoholic neuropathy may be caused by ethanol toxicity or the toxic effects of ethanol metabolites, thiamine deficiency, and a deficiency of other nutrients. How alcohol toxicity affects the peripheral nervous system has not yet been clarified. It is also worth mentioning that the amount of ethanol that may cause clinically significant peripheral neuropathy has not yet been determined as well.

## 4. Optic Neuropathy Induced by Toxic Substances

### 4.1. Methanol, Ethylene Glycol, and Diethylene Glycol

Toxicity induced by methanol, ethylene glycol, and diethylene glycol is caused by their toxic metabolites rather than alcohols themselves [73,74,75].

Methanol might be readily absorbed via the gastrointestinal, respiratory, and skin routes [74]. Ocular damage may be induced by the intrinsic toxicity of formic acid, a metabolite of methanol, whereas the acidosis of formate conceivably accelerates the ocular injury [54,74]. Oral ingestion at levels of 4–15 mL or 3.16–11.85 g/person of pure methanol might cause permanent blindness [74]. The accumulation of formate causes the inhibition of cytochrome oxidase activity and precludes oxygen use by mitochondria, impairing oxidative phosphorylation [75,76]. Cytotoxic effects are associated with a decreased aerobic production of adenosine triphosphate (ATP) and the impairment of ATP-requiring intracellular reactions [75], leading to the interruption of axoplasmic flow, intra-axonal swelling, and optic disc edema [77]. The susceptibility of the optic nerve fibers to formate toxicity might be a result of the existence of fewer mitochondria and lower cytochrome oxidase reserves [78,79]. Clinical manifestations regarding ocular damage include the dimming or blurring of vision, photophobia, defects of the visual field, visual hallucinations, a reduction in visual acuity, and even a complete loss of light perception [80].

Ethylene glycol is readily absorbed from the gastrointestinal tract, while absorption through the skin and lungs is very low [81]. Ethylene glycol is metabolized by a series of oxidative steps, primarily leading to a glycolic acid, the main metabolite causing metabolic acidosis. Glycolic acid is slowly oxidized to glyoxylic acid with subsequent conversion to oxalate, which may be precipitated in the kidney, cerebral blood vessel walls, and other tissues as calcium oxalate crystals [81,82]. However, conceivably, only a small amount of glyoxylic acid is converted to oxalate because glyoxylic acid, as a normal product of hydroxyproline metabolism, is mostly converted to glycine [83]. Clinical presentation typically consists of three phases—neurological, cardiopulmonary, and renal—while ocular manifestations might include diplopia, nystagmus, anisocoria, or decreased visual acuity, leading to optic atrophy [75,82].

Both methanol and ethylene glycol are initially metabolized to their toxic metabolites by the same enzyme—alcohol dehydrogenase (ADH). Thus, treatment primarily relies on the blockage of the formation of these metabolites by ADH [82,84]. Currently, ethanol and fomepizole appear to be antidotes for methanol and ethylene glycol poisoning, presenting significantly higher affinity for ADH compared to methanol and ethylene glycol [84,85,86]. Moreover, therapy is based on general supportive care, the correction of the electrolytes and acidemia, or the use of hemodialysis [86,87]. Nevertheless, despite relevant therapy, methanol poisoning may induce severe and irreversible optic neuropathy [88,89]. Currently, intravenous erythropoietin (EPO) administration appears to be a promising treatment for methanol-induced optic neuropathy, especially when it is combined with a standard treatment [77,78]. EPO is a cytoprotective cytokine that exerts antioxidative and anti-inflammatory effects, reduces neuron cells apoptosis, and exerts neuroprotective and neurodegenerative properties [90,91,92]. Pakravan et al. (2016) showed that patients suffering from methanol optic neuropathy, when treated with a combination of intravenous EPO and high-dose intravenous steroids (reducing optic nerve head edema), exhibited significant functional and structural improvement in vision [78]. Similarly, Pakdel et al. (2018) revealed that intravenous EPO, when used within 3 weeks of methanol ingestion, caused rapid improvement in visual acuity [77].

Regarding diethylene glycol, it is easily absorbed through the gastrointestinal tract and minimally through intact skin, while respiratory absorption is unknown [93]. Moreover, 2-hydroxyethoxyacetic acid and diglycolic acid are the main metabolites responsible for the toxicity, but the key mechanism of diethylene glycol neurotoxicity is not fully elucidated. Clinical features encompass three phases: (1) gastrointestinal with central nervous system features and metabolic acidosis, (2) renal with high anion gap metabolic acidosis, (3) delayed phase with a progressive neurological syndrome, characterized by encephalopathy and various peripheral and cranial neuropathies, including optic neuropathy [94,95,96,97,98]. Treatment relies on the administration of fomepizole and aims to reduce the conversion of diethylene glycol to its toxic metabolites and the use of hemodialysis [73,94,99].

### 4.2. Cobalt, Lead, and Other Heavy Metals

In the past, cobalt (Co) neurotoxicity was observed concerning occupational or iatrogenic (cobalt chloride had been used as a remedy in refractory anemia, nephritis, or infections) exposure. Neurological derangements included, inter alia, progressive bilateral nerve deafness with tinnitus, limb paranesthesia, impaired vibration sense, difficulties in attention, and visual failure leading to optic atrophy [100]. Currently, the main source of Co poisoning remains orthopedic implants, which release metallic ions from Co-containing alloys [100,101]. Apostoli et al. (2013) showed, with respect to the previous studies describing Co- and chromium (Cr)-related toxicity with distinctive ocular and auditory disturbances in patients with a hip prosthesis [102], that rabbits treated with intravenous injections of Co revealed, in histopathological findings, severe cochlear and retinal ganglion cell depletion, damage of the optic nerve, and loss of the sensory cochlear hair cells [102]. Those authors indicated that the animals had been exposed to a high dose of Co and Cr, alone or in combination, but no evidence of clinical and pathological alterations after Cr administration was found, while the Co group showed distinguishing abnormalities on the auditory and optic systems. The severity of alterations depended on Co dosages and the time of Co exposure [102]. The treatment of Co poisoning is based on the removal of the Co source and N-acetylcysteine (NAC) chelation therapy [101,103]. NAC may also counteract retinal ganglion cell apoptosis, and it might exert neuroprotective effects [101,104].

Lead (Pb) intoxication affects the optic nerve, retina, radiation, visual cortex, lens, or extra- and intra-ocular muscles, although ocular manifestations usually occur at a later stage of disease, concerning 1% to 2% of Pb poisoning cases [105]. Baghdassarian (1968) reported a case of bilateral optic neuropathy in a patient who presented with a gradual decrease in vision in both eyes associated with retrobulbar pain, and they had laboratory evidence of systemic Pb poisoning without other clinical manifestations. The complete improvement of vision after favorable therapy with penicillamine as a chelating agent was observed [105]. Abri Aghdam et al. (2019) described a case of Pb-induced painless optic neuropathy presenting with bilateral hemorrhagic optic disc swelling in a patient who also presented with ataxia and paresthesia of the lower extremities [106]. Patel and Athawale (2005) reported a case of an 11-month-old girl who presented with acute Pb poisoning encephalopathy resulting in optic neuropathy. Those authors assumed that the combination of the raised intracranial pressure and anemia in the course of acute lead encephalopathy resulted in ischemic optic neuropathy [107]. Chronic Pb exposure results in decreased retinal nerve fiber layer, macular, and choroidal thickness [108]. Ocular symptoms were also observed after mercury, thallium, arsenic, or gallium exposure [109,110,111]. Regarding mercury (Hg) poisoning, Cavalleri et al. (1995) reported that occupational exposure to inorganic elemental Hg vapor induces a dose-related color vision loss [112]. In the following study, Cavalleri and Gobba (1998) showed that after the improvement of working conditions and a reduction of Hg vapor exposure, color perception was significantly improved, indicating that the alterations are reversible [113]. Conversely, Feitosa-Santana et al. (2007), during an evaluation of other people who were occupationally exposed to Hg vapor, showed that color discrimination losses were present many years after the end of exposure to elemental Hg, suggesting that alterations might be irreversible [114]. Similarly, organic methylmercury exposure may induce abnormal color vision, a constriction of the visual field, poor night vision, or sudden bilateral blindness [115,116]. Abnormal color vision might be the first clinical sign of optic neuropathy [112].

Regarding thallium (Tl) intoxication, severe bilateral optic neuritis was notified in 25% of acute thallium poisoning cases and in almost all cases of chronic poisoning [109]. Functional changes associated with optic nerve damage include impaired visual acuity, abnormal color vision (especially tritanomaly), the impairment of contrast sensitivity, and central or cecocentral scotomas [117].

Regarding arsenic (As) toxicity, pentavalent arsenical (particularly tryparasamide) administration might cause optic neuritis with sudden (within 2 to 12 h after ingestion) impairment of vision [111]. Thery et al. (2008) described a case of As-related optic neuropathy in a patient who had been treated with arsenic due to acute promyelocytic leukemia relapse. The ocular alterations had appeared after 5 months of treatment and regressed up to 6 months after As interruption [118]. Freund et al. described a case of a slowly progressive bilateral symmetric optic neuropathy caused by chronic As exposure in a patient who presented with insidiously progressing visual acuity loss. It was established that the patient had spent almost every weekend for the past 28 years at a country cottage, where the water sourced from the local well was contaminated by naturally occurring As [119]. As toxicity appears to be time- and dose-dependent, while the lethal dose is estimated to be about 0.6 mg/kg/day [48,118].

Regarding gallium (Ga) toxicity, gallium nitrate may cause optic neuropathy by interference with the function of optic nerve oligodendrocytes. Gallium nitrate binds to serum transferrin, and it is transported into cells by transferrin receptors, impairing iron transport and metabolism and leading to cell death, particularly in rapidly dividing cells. Transferrin receptors are present on the optic nerve oligodendrocytes, and thereby gallium nitrate could damage the optic nerves [120].

### 4.3. Drugs

It is now well established that a wide range of medications might cause toxic optic neuropathy in a way that is dose- and duration-dependent [54]. The drugs responsible for optic neuropathy include antimicrobial agents (linezolid, ciprofloxacin, cimetidine, and chloramphenicol), antituberculotic drugs (isoniazid and ethambutol), halogenated hydroquinolones, benzofuran derivatives (amiodarone), antiepileptic agents (vigabatrin), cGMP-specific phosphodiesterase type 5 inhibitors (sildenafil, tadalafil, and vardenafil), methotrexate, cisplatin, carboplatin, 5-fluorouracil, vincristine, cyclosporine, tamoxifen, and disulfiram, as well as tumor necrosis factor-alpha (TNF-α) antagonists (etanercept, infliximab, and adalimumab) [54,121,122]. Cases of optic neuritis as an adverse event of immune checkpoint inhibitor (ICI) ingestion have also been reported, most commonly with ipilimumab, a CTLA-4 inhibitor. Further, ICIs such as PD-1 inhibitors (nivolumab and pembrolizumab) and PD-L1 inhibitors (atezolizumab or durvalumab) might cause optic neuropathy [123,124].

Optic neuropathy is also a well-known and quite common complication that might be a result of ethambutol intake. The prevalence of ethambutol-induced optic neuropathy (EON) amongst tuberculosis patients is estimated to be 1–2% [125]; its occurrence is also dose-dependent. The neurotoxicity of ethambutol on optic nerves is yet not clearly established; it is assumed that metal chelating effects of ethambutol might be responsible for that. Further, prolonged usage of ethambutol is also associated with deficiencies in vitamins E and B12, which additionally exacerbate the symptoms of optic neuropathy. Most common symptoms of EON include the painless loss of central vision and the presence of cecocentral scotomas in the field of vision [126]. The majority of patients present with bilateral, symmetric loss of central visual acuity as well as dyschromatopsia [125]. Usually, the loss of color vision (primarily the loss of red and green color perception) is the first sign of EON.

### 4.4. Other Toxic Substances

Toxic optic neuropathy may be also caused by carbon monoxide (CO), industrial solvents (toluene, styrene, tetrachloroethylene, carbon disulfide, n-hexane, and solvent mixtures), or organophosphate pesticides [54,127,128]. CO poisoning may present in ophthalmologic examination with decreased visual acuity, morphological changes, and delays in both the flash and pattern VEPs (visual evoked potentials), abnormal pattern ERG (electroretinogram), and pale optic disc in fundoscopy. The mechanism underlying CO-induced toxic optic neuropathy may be similar to that in tobacco amblyopia. CO-induced optic nerve damage could be, at least partially, reversible, and early treatment with hydroxocobalamin may be beneficial as in cases of tobacco amblyopia [127]. Furthermore, CO poisoning may precipitate the clinical expression of LHON [129]. Occupational exposure to solvents or some other chemicals, such as organophosphate pesticides, may lead to color vision loss that is usually subclinical, and thereby vision testing may be favorable in the evaluation of early neurotoxicity of chemicals in exposed workers [129]. The impairment of color discrimination was notified in cases of exposure to environmental levels lower than the occupational limits proposed [130].

## 5. Nutritional Deficiencies as a Trigger of Optic Neuropathy

Optic nerve dysfunction could be triggered by nutritional deficiencies, toxins, and drugs. Common triggers in this category include methanol, amiodarone, tobacco, and ethanol [131]. Just like inherited optic neuropathies, optic neuropathies induced by nutritional deficiencies are characterized by selective maculo-papilar bundle involvement, resulting in central or cecocentral scotomas. At least 1% of patients may experience optic neuropathy caused by ethambutol [132]. Patients who are deficient in zinc (Zn) are highly vulnerable to optic neuropathy [133]. Symptoms usually begin between 2 and 8 months after drug administration. The patient may have very subtle pupillary abnormalities. In some cases, there may be a need for a visual evoked potential to confirm the diagnosis.

The condition tobacco–alcohol amblyopia is also worth mentioning. This condition may be due to the toxic effect of tobacco as well as a state of nutritional deficiency. Because most patients who abuse tobacco and alcohol do not have optic neuropathy, the mechanism may not necessarily be a direct insult to the optic nerve. Nutritional deficiency and genetic factors may make the patient more susceptible to optic neuropathy. A deficiency of nutrients such as riboflavin, thiamine, folate, B6, and B12 is associated with optic neuropathy [134]. Patients following ketogenic, high-protein, and low-carb diets have a high risk of thiamine deficiency. Additionally, patients whose gastrointestinal tract has been surgically operated on may develop optic neuropathy as a result of interference with vitamin B12 absorption.

## 6. Treatment Options

The therapeutic options in the treatment of optic neuropathy strictly depend on the causative factor. Optic neuropathy, induced by nutritional deficiencies, is treated with appropriate supplementation, so it is crucial to accurately determine the missing vitamins and elements. The main players behind the development of nutritional optic neuropathy are nutrients that condition the proper function of the mitochondria, mainly copper and B vitamins, with the greatest importance being vitamin B12 (cobalamin), vitamin B9 (folic acid), and vitamin B1 (thiamine) [135]. The British Society of Hematology recommends treating vitamin B12 deficiency with a daily intramuscular injection of 1000 lg of hydroxocobalamin until improvement is achieved [136]. The therapy is then maintained with a single intramuscular injection of 1000 µg of hydroxocobalamin once every two months. The guidelines state that treatment should be instituted as soon as possible to avoid the persistence of neurological disabilities. When treatment is started, improvement usually occurs after 6 weeks to 6 months [137]. If a vitamin B9 deficiency is coexisting, it should be replaced first to prevent the subacute degeneration of the spinal cord [137,138]. The therapeutic dose of vitamin B9 depends on the cause of its occurrence; the guidelines do not emphasize the exact supply in the case of neuropathy, while in the case of nutritional deficiencies and the occurrence of megaloblastic anemia, an oral intake of 5 mg of folic acid/day for a period of 4 months is recommended [136]. The appropriate daily amount of copper has not been accurately established, and it fluctuates between 1.5 and 3 mg orally per day, with higher doses which are gradually tapered down [139]. In a more severe course, intravenous therapy is also possible [139,140]. Vitamin B1 can be administered intravenously or intramuscularly in a dose of 100 mg daily for 2 weeks, and then continued as an oral supplementation with a group B vitamin complex [60]. For Wernicke’s encephalopathy, at least 500 mg of thiamine should be given three times a day for 2–3 days [141]. As for tobacco–alcohol optic neuropathy, it has a different prognosis depending on the degree and duration of exposure to alcohol and tobacco, and the duration of vision impairment until diagnosis [58]. The most important thing in this case is to stop drinking alcohol and smoking, to eat a well-balanced diet and to compensate for vitamin deficiencies. As mentioned earlier, methanol poisoning can lead to complete blindness. The effects of methanol poisoning are difficult to predict—some patients experience improvement in vision, while others experience worsening vision over time [142]. There are several human clinical studies suggesting the utility of recombinant human erythropoietin (EPO), either intravenously or in combination with systemic steroid therapy, where the addition of EPO significantly improved the treatment effect [77,78]. Erythropoietin is a hormone mostly produced by adult kidneys that has been shown to protect the cells of the retina from excessive oxidation and exposure to strong light. This hormone exhibits antioxidant, anti-inflammatory, neuroprotective, angiogenic, and anti-apoptotic effects [143]. There is also a report that the improvement in vision is only transient after the intravenous administration of EPO in combination with methylprednisone, and a single study has shown that the efficacy of EPO in methanol-induced optic neuropathy was not observed in the late cases of the disease [142,143,144].

Due to the multitude of medicinal substances capable of inducing toxic optic neuropathy, only ethambutol will be discussed in this publication, as it is the drug most often causing optic neuropathy, whereby the incidence of this disease is up to 100,000 cases per year [145]. Ocular side effects are believed to be dose-dependent, with most patients developing neuropathy at 60–100 mg/kg/day, although this is possible even at ≤15 mg/kg/day [146]. In addition, patients with kidney disease should not be treated with ethambutol, as this drug is mainly excreted via the kidneys and accumulates in the body when the kidney disease is present [147]. Discontinuation of the drug usually results in visual improvement [126,148], although some reports indicate that visual disability persists [149,150], as well as progressive structural damage in the absence of clinical symptoms [151]. It is also noted that asymptomatic patients treated with ethambutol should undergo monthly screening of at least visual acuity and Amsler grid testing to detect impairments promptly [125].

## 7. Conclusions

This overview summarizes the current state of knowledge regarding the toxic and nutritional etiopathogenesis of optic neuropathies. Optic neuropathies constitute a group of conditions with several causes, including both genetic (Leber’s hereditary optic neuropathy) and acquired (drugs, nutritional deficiencies, alcohol, tobacco, ischemia, environmental toxins such as organophosphate pesticides, carbon dioxide, industrial solvents, and heavy metals such as cobalt, lead, mercury, thallium, arsenic ang gallium). Ethanol, despite some health benefits (cardioprotective effect while consumed in small amounts) is toxic to neurons both directly and indirectly (nutritional deficiencies, malnutrition, genetic factors, and toxic metabolites). The causes of alcohol-induced optic neuropathy are unknown, and its pathogenesis is multifactorial. It is assumed that the main cause of neuropathy may be nutritional deficiencies, mainly vitamin B12 deficiency. The term “alcohol–tobacco” optic neuropathy is controversial because most people who abuse alcohol and smoke cigarettes do not develop neuropathy. Scientists are, therefore, inclined to believe that an additional factor (genetic or nutritional) is needed to trigger visual impairment. The treatment of such neuropathy consists of compensating for vitamin deficiencies and alcohol and tobacco cessation. When it comes to the genetic causes of optic neuropathy, symptoms come from a mutation within the mitochondrial DNA and NADH-encoding dehydrogenase, which impairs the synthesis of ATP and causes the formation of oxygen free radicals, destroying retinal ganglion cells. The ophthalmologists should be aware of numerous causes of optic neuropathies, including disturbed nutritional status, as well as the toxic etiopathology induced by the chronic alcohol consumption or the accumulation of heavy metals that disturb the physiology of the eye.

## Data Availability

Not applicable.

## Abbreviations

ADH	alcohol dehydrogenase
EON	ethambutol-induced optic neuropathy
EPO	intravenous erythropoietin
LHON	Leber hereditary optic neuropathy
TON	toxic optic neuropathy
WHO	World Health Organization
